# Perceived exercise habits of individuals with Parkinson’s disease living in the community

**DOI:** 10.1016/j.prdoa.2021.100127

**Published:** 2021-12-17

**Authors:** Jordana Lockwich, Kate Schwartzkopf-Phifer, Camille Skubik-Peplaski, Richard D. Andreatta, Patrick Kitzman

**Affiliations:** a University of Evansville, Evansville IN 47722, USA; b University of Kentucky, Lexington KY 40506, USA

**Keywords:** Parkinson’s disease, Gait, Exercise, Intensity

## Abstract

**Context:**

Exercise has been shown to improve gait in individuals with Parkinson’s disease (PD). Stepping practice at higher intensity levels has been suggested as a beneficial treatment option to improve gait in the neurological population. Unfortunately, this mode is poorly understood and underutilized within the PD population. Information on what individuals with PD are doing for exercise would be beneficial to help tailor exercise programs to improve gait and provide exercise options in the community for intensity-based exercise.

**Objective:**

To investigate the current exercise habits of individuals living with PD in the community aimed at improving walking and to understand the impact of perceived intensity on daily exercise practices.

**Design, setting, participants:**

One hundred thirty-eight individuals with PD living in the community were surveyed online regarding their current exercise habits.

**Main outcome measure:**

A total of 22 questions aimed to understand exercise selection, focus, and perceived intensity. Questions asked basic demographic, symptom presentation and management of disease related symptoms that were present while living with PD. Exercise questions focused understanding participants current function level, practice exercise habits and perceived levels of exercise intensity during daily routines.

**Results:**

Of the 138 individuals surveyed for this preliminary study, eighty-seven percent of individuals with PD participated in exercise with seventy-five percent choosing walking as a mode for exercise. Sixty-five percent of the respondents noted that despite exercise, their walking speed and endurance has worsened since diagnosis. Eighty-one percent perceived exercising at moderate intensity levels, however little provocation of intensity symptoms was noted.

**Conclusion:**

Our preliminary study survey results suggest that individuals with PD are exercising but not at high enough intensity levels to promote improvements in gait performance. Individuals with PD may need to be pushed at higher intensity levels, beyond their voluntary limits, to induce gait performance changes. These findings can provide a foundation for future fitness interventions within this population to target improving gait.

## 1 Introduction

PD is the second most common degenerative disease in older adults, affecting 1% − 2% of individuals above 60 years old [1]. According to the National Parkinson’s Foundation, 1 million people currently have PD in the United States and 4–6 million people have been diagnosed worldwide [2]. It is estimated that the PD prevalence will continue to rise and there will be over 60,000 new cases in the United States by 2030 [2]. PD is an incurable neurodegenerative disease that is characterized by cardinal motor features including bradykinesia, resting tremors, muscular rigidity and postural instability [3]. These classic motor symptoms affect overall gait performance and therefore need to be addressed to improve functional mobility [4].

Gait problems are one of the most debilitating consequences of Parkinson’s disease (PD) [5]. With PD progression there is a complete inability to control normal movement patterns that were once automatic. Walking, a previously automatic motor task, is directly affected and limits participation in mobility activities. These gait problems are immense and are becoming a global problem with the rise in PD prevalence [2].

Fortunately, exercise targeting gait performance for individuals with PD is neuroprotective despite the progressive consequences of this neurodegenerative disorder [6]. Breaking down gait into biomechanical subcomponents allows for targeted interventions to improve functional mobility. These subcomponents include limb swing advancement, propulsion, stance control and lateral stability [7]. Individuals with PD often have difficulty with the propulsion subcomponent and demonstrate slow gait speed, shorter step lengths and poor adaptability to variable environments. These propulsion deficits can impact one’s ability to participate in community activities. Luckily, exercise targeting improving cadence has been identified as a key variable to improving propulsion gait deficits in PD [8].

Furthermore, stepping practice at higher intensities with individuals with PD targets improving walking performance whereas lower intensity, goal directed programs may not [9], [10], [11]. Forced exercise at higher intensities when compared to voluntary exercise produces global improvements in motor function on clinical rating scales [11]. When compared to a control group or usual care, intensity-based training has improved disease severity motor scores, oxygen consumption maximum levels, and cadence numbers as well as specific gait parameters such as variability, step length and arm swing [12], [13]. Practicing stepping at high-intensity levels of 80% of one’s heart rate maximum (HRmax) or higher was found to be well tolerated with minimal adverse effects [13]. High intensity practice even produced a reduction in disease severity motor scores where there was no demonstrated change with individuals that trained at a moderate intensity level [13]. Improvements on disease severity has been well documented but little has been reported on gait metrics at high levels of intensity-based exercise within the PD population. The key difference in the findings support the use of high-intensity exercise to promote adaptive changes in PD outcomes [14]. However, the use of high intensity stepping exercise is underutilized in current community programs, and the proper delivery of these interventions is unclear.

Community programs available for individuals with PD consisted of group-based exercise that focus on activities such as cycling, boxing, flexibility and strength. Such programs have shown to improve PD severity rating scores [11], [14], [15], [16] but it is unclear about how much effort the participant is actually putting forth. Though these programs tend to provide a well-tolerated environment to improve motor impairments of PD, there is little emphasis on measured intensity and task specific walking practice. Individuals with PD need more options in the community to improve their walking and there are no current options available.

It is unknown if individuals with PD that have gait problems are choosing the correct type of exercise to improve their walking ability. The goal of this research to gain knowledge that will help provide future education about choosing the most beneficial type of exercise to counteract disease progression impairments that are present. Also, despite the known benefits of high intensity exercise, little is known about how much effort individuals with PD are exercising. This understanding of perceived effort that is being utilized during current exercise practice will provide reflection and guidance on the adjustments needed for the exercise prescription. Finally, the results of this survey will be used to tailor future walking exercise programs targeting high intensity stepping practice for the PD community to improve walking.

Before additional community programs can be established, there is a critical need to understand what individuals with PD living in the community are currently doing for exercise. Therefore, the purpose of this survey aims to investigate the current exercise habits and perceived intensities of individuals with PD. We hypothesize that individuals with PD are not exercising at high enough intensities to induce changes in their walking performance.

## 2 Methods

### 2.1 Study Design

A 22-question self-administered survey was emailed to a community-based sample of individuals with PD living in the community. This project was approved by the University of Kentucky and the University of Evansville Institutional Review Boards.

### 2.2 Study population & sample size

A total of 202 individuals with PD were recruited from community based organizations in the Midwest region that served this rare neurological population. Organizations included local support groups, exercise programs and local hospitals. There is currently no national database that provides a PD population total in this US and the prevalence of PD varies across the country. However, the midwest area provides a unique opportunity for investigating the PD population whereas the incidence was found to be 2-10x greater when compared to the western and southern counties [17].

Identified members of the PD community were granted access to the online survey via a secure link sent from Qualtrics software programing, a widely used tool for survey research. Agreeable participants were asked to complete the survey if they had PD and were given access via a secured online link after acknowledgment of an informed consent was signed.

### 2.3 Study outcomes

The survey was developed by the PI and a panel of clinicians and researchers. The initial survey was piloted to individuals with PD and clinicians who treat individuals with PD and was revised based on their feedback. The final survey consisted of 22 questions that were separated in four blocks of interest (see Table 1). The first block asked basic demographics questions and aimed to understand the individual’s current symptom presentation and management strategies for their PD symptoms. The second block included questions regarding their current level of function and walking ability after being diagnosed with PD. The last two blocks aimed to understand current exercise habits and perceived intensity levels. Intensity of exercise was defined as a presence of symptoms that include sweating, shortness of breath, fatigue, muscle soreness, and early termination of exercise. [18] Level of intensity during exercise was categorized as either being light, moderate or vigorous which was defined based on the Borg Rated Perceived Exertion Scale parameters [19]. Questions in the intensity block emphasized understanding how intense individuals were exercising and if intensity symptoms were produced with routine habits.

**Table 1:** Survey Questions.

Demographic Block	Current Level of Function Block
• How old are you? • How old were you when you were diagnosed with Parkinson’s disease? • What is your gender? • What is the highest level of education that you have completed? • What is your current living situation? • Has your Parkinson’s Disease been staged? If so, which stage would you classify your symptoms? • Do you take medications for your Parkinson’s Disease? • Have you undergone deep brain stimulation for your Parkinson’s disease?	• Do you need any physical help from another person during your typical day? • Describe how you walk on a typical day • Do you feel you need to improve on balance, endurance, strength, flexibility or something else the most? • Describe you walking – better or worse since you were diagnosed?

**Current Exercise Block**	**Exercise Intensity Block**
• After you were diagnosed, did any of the following (Lack of knowledge on what to do for exercise, did not have adequate assistance, limited transportation, fear/anxiety, equipment availability) impact your ability to exercise? • Where do you work out most of the time? • Who do you work out with most of the time? • What do you choose to work on when you work out? • How long do you typically exercise? • How many times do you typically exercise per week? • Do you walk for exercise?	• My exercise intensity is light, moderate, Vigorous • How likely are you too? o sweat during your exercise? o get short of breath during your exercise? o get tired during your exercise? o monitor your heart rate during o your exercise? o get sore over the next day or two after your exercise? o end your exercise early due to feeling tired, short of breath, sweating and/or soreness?

### 2.4 Statistical analysis

All responses were anonymous and no identified information was collected. Descriptive summaries were generated in the Qualtrics software and means/standard deviations were reported for continuous variables. Categorical variables such as gender and patient characteristic reported in the survey were summarized as frequencies and percentages. Differences in disease severity were compared using a Kruskal-Wallis analysis, as appropriate.

## 3 Results

### 3.1 Study population

Out of the 202 questionnaires successfully distributed to identified individuals with PD living in the community, 138 were returned, yielding a response rate of 68%. Most respondents were older than 60 years (91.7%, n = 121), the majority were male (n = 84), and most had completed some form of higher education (92.1%, n = 122). Nearly half of the participants (n = 62) have been living with PD for at least 6 years and most described their PD symptoms as being mild to moderate on the disease rating scale (n = 111). Few individuals had received deep brain stimulation for management of their PD symptoms (9%, n = 12) but most utilized medication (94%, n = 124). Mild, moderate, and severe PD was represented in our respondents for this study. Though the majority of the respondents reported mild (54%, n = 57) to moderate (30%, n = 32) PD severity, there was a small number of individuals that reported living with severe progression of the disease (15%, n = 16). Table 2 describes the demographic characteristics of the respondents and their clinical descriptions.

**Table 2:** Demographic and Clinical Characteristics of PD Survey Respondents.

Demographics	Percentage
Age > 60y	91.7
Age > 70y	57.0
Gender (male)	63.6
Education (at least Bachelors or higher)	76.5
Lives with support	85.6
**PD Diagnosis**	
2–4 y with PD	30.3
6 + y with PD	47.1
**PD Severity**	
H&Y 1–2 (mild)	47.8
H&Y 3–4 (moderate)	45.5
H&Y 5–6 (advanced)	6.8
**Management of PD symptoms**	
Uses Medication	93.9
Number of Years on Medication (Mean ± SD)	6.73 ± 5.480
Has had Deep Brain Simulation (DBS)	10.0

### 3.2 Disease severity

Survey respondents, regardless of disease severity, acknowledged participating in some form of exercise during their daily routines. Though Kruskal-Wallis analysis revealed there was a difference in the reported symptoms of perceived intensity when comparing different PD severity levels. (see Table 3) Individuals with mild PD were more likely not to report if they experienced sweating during exercise [H(2) = 8.368, P = .015] or had to stop exercise because of intensity symptoms. [H(2) = 9.727, P = .008] Whereas individuals with severe PD were in fact more likely to report these symptoms when participating in daily exercise routines. Post hoc analysis revealed that there were no significant differences found in perceived exercise intensity levels, duration, and dosage across disease severity.

**Table 3:** Kruskal-Wallis Analysis and Mean Ranks results of PD severity levels and reporting intensity symptoms with exercise. Symptoms of sweating and stopping exercise were found to be significant between individuals with mild PD and severe PD.

	**PD Severity**			
	Mild (n = 57)	Moderate (n = 32)	Severe (n = 16)	P-Value
**Intensity Symptoms**				
Exercise Intensity	53.47	54.36	48.59	0.703
Heart Rate	54.03	49.59	56.16	0.566
**Sweating**	**59.41**	**49.70**	**36.75**	**0.015**
Shortness of Breath	51.65	47.95	67.91	0.067
Fatigue	51.39	49.98	64.78	0.198
Soreness	54.80	47.64	57.31	0.339
**Stop Exercise**	**47.55**	**53.75**	**70.91**	**0.008**

### 3.3 Walking habits & changes

**Fig. 1:**
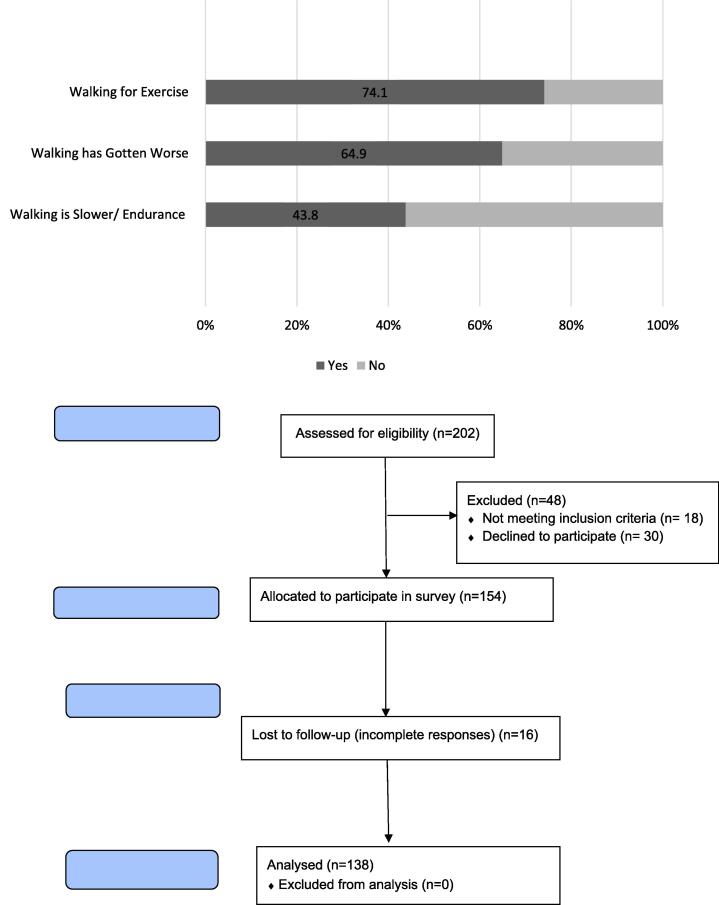
Current Walking Habits and Changes Reported with PD.

Most individuals described their current level of functional mobility as independent which was defined as the ability to walk on their own without the physical assistance of another person, regardless of assistive device (71%, n = 94; Fig. 1).

The majority of the survey respondents participated in some form of exercise (87%, n = 113) including both individual exercise and group-based exercise programs. Seventy-five percent of individuals reported when choosing what type of exercise to participate in, they used walking for exercise over other modes of activity (n = 92). The most reported environment to practice walking was outside of the home and in the community that consisted of changes in surface types that challenged balance (66%, n = 91). Few barriers to exercising were reported and our respondents felt that there was nothing impeding their ability to work out and routinely participated in exercise in their daily habits.

However, when asked about how PD had affected their walking, 65% (n = 85) reported that their walking has gotten worse since their diagnosis despite participating in routine exercise practice. Decreased speed (37%, n = 51) and endurance (31%, n = 42) were two of the most common reported gait impairments that were attributed to the decline in functional mobility.

### 3.4 Perceived exercise intensity

On average, individuals exercised at least 3 days a week (42% n = 46) with some reporting exercising at least 5 times per week (41%, n = 45). Most exercise sessions lasted at least 45 min (24%, n = 27) with the majority being close to an hour in length. (55%, n = 61) When asked about how challenging their exercise was, most individuals answered that they felt that they were exercising at a moderate intensity level (75%, n = 81). Moderate intensity exercise in this study was defined as breathing heavy but still able to hold a short conversation while exercising [19].

**Fig. 2:**
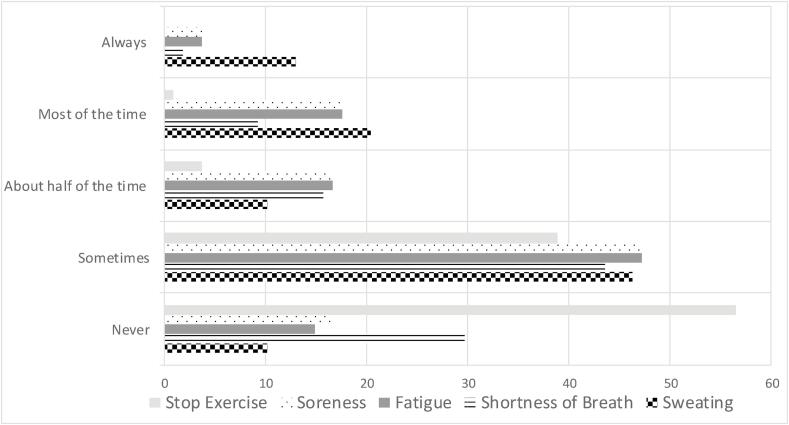
Report of the likelihood of symptoms of intensity during routine exercise (5 point Likert scale).

**Fig. 3:**
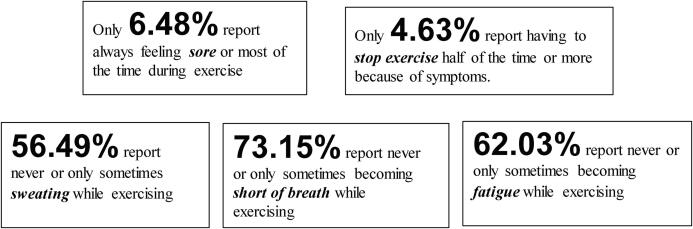
Summary of Exercise Intensity Symptoms Reported.

Though individuals reported perceived exercise intensities at a moderate level, reported symptoms did not support this finding. When asked to rate how likely it was for them to display signs and symptoms of intensity during common exercise routines, over half of individuals reported never or only sometimes displaying symptoms of sweating (56%, n = 61), shortness of breath (73%, n-100) and muscle soreness (5%, n = 7) after exercise. Only one respondent had to end exercise early (1%, n = 1) because of these challenging symptoms of perceived exercise intensity. Fig. 2, Fig. 3 describe the symptom provocation of the respondents during exercise.

## 4 Discussion

The objective of our survey was to investigate the current exercise habits of individuals with PD and understand the level of perceived intensity that exercise was being practiced.

Despite the progressive nature of PD, exercise is both neuroprotective and a positive contributor to improving mobility. Offsetting the sequence of this neurodegenerative disorder, exercise has been shown to improve multiple aspects of functional mobility, including gait [20], [21], balance [22], [23], [24] flexibility [22] and strength 35,33. Maintaining an active lifestyle a lone has been linked to the preservation of skeletal muscle mass strength regardless of the disease progression. [25] Reducing fall risk ([21], [24], [26] Nonmotor symptoms of PD can also be impacted by exercise with improvements reported and quality of life in this population

However, barriers to exercise still exist including low outcome expectation, fear of falling, lack of time as well as poor motivation. [27] Individuals with PD have reduced levels of physical activity when compared to their healthy peers.[28] Avoiding a sedentary lifestyle and engaging in daily physical activity can improve motor symptoms of PD.[29] Specific exercise, that is planned, structured and repetitive with the set intention of making improvements in goals, such as walking, is recommended for individuals with PD to improve their health and well-being. [30]

Overall, our results demonstrate that individuals with PD are motivated to participate in exercise opportunities to manage symptoms of this progressive disease across the spectrum of PD. Regardless of severity level, the majority of respondents with PD are active and participate in some form of exercise at perceived moderate intensity levels. Based on the small sample of this study, results indicated that severity level may not impede the motivation or desire to exercise. Individuals with mild to moderate PD reported exercising at the same level of perceived intensity and duration of those individuals that had severe PD. Differences were reported however in intensity symptom experience. Individuals with mild to moderate PD were less likely to experience intensity-based symptoms during exercise and did not report it as frequently. Whereas individuals with severe PD were more likely to experience symptoms of intensity and report needing to stop exercise early. These results may provide information on proper dosage and how interventions should be delivered at different levels of disease progression. Individuals with mild to moderate PD may be able to be pushed at higher levels of intensity where individuals with severe PD can workout at the same intensity level but may require rest breaks and modifications to reduce intensity symptoms early on. Regardless of disease severity level, intensity driven exercise seems to be a great option to combat PD symptoms [31].

Despite practicing in daily exercise routines, the majority of respondents still noted that their walking has continued to worsen since diagnosis. Degradation of walking, decreased walking speed and poor endurance were the most reported gait deficits by respondents, even with routine daily exercise practice. This realization may indicate that selected exercise practices are not directly targeting gait impairments that are present. Most community-based PD exercise programs that are available aim to improve overall generalized weakness, endurance and mobility. But to our knowledge, there are no specific programs that focus on walking directly. Outlined neurological principles advocate for task specific, repetitive and salient practice to induce neuroplastic changes in the brain [32]. Based on the results of this study, walking is absolutely a salient activity to individuals with PD. Therefore, individuals who want to improve walking should have opportunities to practice walking.

Intensity of exercise is also a key component to induce neuroplastic changes [32]. It was surprising to discover that the participants in our study perceived that they work out at challenging intensities. The results of this preliminary study indicate that individuals with PD may not be exercising at challenging enough intensities to produce symptoms that are consistent with moderate to high intensity exercise. Typically, if someone is to participate in challenging exercise, they will produce some level of intensity symptoms that may include either sweating or becoming short of breath [19]. This was an unlikely occurrence with our participants despite reporting working at higher perceived intensity levels. Research suggests that intensity-based stepping exercise is beneficial for improving biomechanical gait impairments and decreasing disease severity in PD. Schenkman et al. practiced stepping at high intensity (80% of an individual’s heart rate maximum) and reported a reduction in PD severity scores compared to individuals that worked at moderate intensity levels [60–80% heart rate maximum)[13]. When compared to a control group or usual care, another study found that intensity-based training has improved disease motor scores, oxygen consumption (Vo2 max), cadence, and specific biomechanical gait parameters such as variability, step length and arm swing [11]. These results suggest that individuals with PD may need to be pushed at higher intensity levels beyond their voluntary limits at rates sufficient enough to induce gait performance changes. Improvements in disease severity and gait biomechanics has been well documented with intensity-based exercise but little has been reported on clinical measures of gait propulsion outcomes.

The mismatch between actual exercise habits and perceived levels of intensity may be due to lack of awareness and education on what type of exercise one should be participating in to improve their walking ability in the community. There is clearly a need for more education regarding intensity levels of exercise and the benefits of pushing oneself past voluntary limits to improve gait within the PD population. The results of this survey demonstrate the need for exercise options that target high intensity exercise with emphasis on improving walking in the PD population.

*Study Limitations:* Though this preliminary study provided information regarding exercise practices, there are limitations of this current study that are worthy of note. Survey data was obtained through an online platform and individuals that did not have access to the internet were not included, resulting in selection bias. These excluded individuals could have added more variability to our sample, resulting in greater generalizability to the PD population as well as more in depth knowledge about current exercise habits for individuals with PD. Also, generalizability of our results is limited given the heterogeneity of the subjects, as 90% of the respondents were male, over 60 years old, and at a mild to moderate stage of the disease. Finally, given that this was a self report, only perceived exercise intensity levels were analyzed and true intensity measures were not collected. In the future, assessing direct intensity measures this could give more insight into common exercise habits of those individuals living with Parkinson’s disease.

In conclusion, our survey results have demonstrated that individuals with PD are exercising regularly and using exercise as a means to combat progressive symptoms. However, they are still reporting worsening gait problems despite routine exercise habits practiced. Our results suggest that individuals with PD are not exercising at challenging enough intensity levels that are sufficient enough to induce gait performance changes.

Individuals with PD need more education and guidelines for options that include task specific exercise to improve walking performance. Though there are many options for individuals with PD to exercise in the community, to our knowledge there are no structured walking programs available. Future studies will aim to utilize the results of this survey and tailor a walking program with goals to improve overall gait within the PD community.

This research has been registered at https://clinicaltrials.gov/ with a reference number of: NCT0501117.

This research did not receive any specific grant from funding agencies in the public, commercial or not-for-profit sectors.

### CRediT authorship contribution statement

**Jordana Lockwich:** Conceptualization, Writing – original draft. **Kate Schwartzkopf-Phifer:** Conceptualization, Writing – review & editing. **Camille Skubik-Peplaski:** Methodology, Supervision. **Richard D. Andreatta:** Methodology, Supervision. **Patrick Kitzman:** Methodology, Supervision, Investigation.

## Declaration of Competing Interest

The authors declare that they have no known competing financial interests or personal relationships that could have appeared to influence the work reported in this paper.
